# Amyloid-β oligomers unveil a novel primate model of sporadic Alzheimer's disease

**DOI:** 10.3389/fnins.2015.00047

**Published:** 2015-03-16

**Authors:** Joseph D. Jebelli, Thomas M. Piers

**Affiliations:** ^1^Department of Neurology, University of WashingtonSeattle, WA, USA; ^2^Therapeutic Innovation Group, Institute of Neurology, University College LondonLondon, UK

**Keywords:** Alzheimer's disease, amyloid-beta oligomers, primate models, mouse models, tau phosphorylation

The development and characterization of Alzheimer's disease (AD) animal models that faithfully recapitulate key components of the pathogenic process has been a major research focus for nearly a quarter of a century. Neuropathologically, AD is characterized by the accumulation of extracellular amyloid-β (Aβ) plaques, intracellular neurofibrillary tangles (NFTs), synapse loss, glial cell activation, and neuronal death. The creation of genetically altered rodent models has significantly advanced our understanding of AD pathogenesis, and many novel therapeutic drugs are currently in clinical trials as a result (LaFerla and Green, 2012). Transgenic mouse models most commonly harbor mutations in APP and/or presenillin 1—gene mutations that are linked to early onset familial forms of AD (fAD). Nevertheless, the vast majority of AD cases (≈95%) are sporadic (sAD), with an unknown genetic etiology and underlying cause. Consequently, fAD models pose a number of problems for the translation of preclinical drug studies to human clinical trails.

In a recent study published in *The Journal of Neuroscience*, Forny-Germano et al. (2014) injected soluble Aβ oligomers (AβOs) into the lateral ventricle of adult cynomolgus macaques. These primates hold great potential as models of AD due to their biological proximity to humans, relatively large brains, complex behavior, and capacity to generate human-sequence Aβ that deposits as plaques in the brain with aging (Heuer et al., 2012). The authors demonstrated that AβOs diffuse and accumulate in memory-related brain regions, causing tau phosphorylation, NFT formation, synapse pathology, and astrocyte and microglial activation. This effect was seen in the absence of extracellular amyloid plaques and replicated using 3-month-old Wistar rats in which a similar profile of pathology was observed. In accordance with studies using fAD transgenic mice (Tomiyama et al., 2010), AβOs did not trigger acute neuronal cell death; however, unlike fAD mice the macaques displayed widespread tau pathology. This phenomenon is only seen in transgenic mice if a mutation unrelated to human AD is introduced into the tau gene (Lewis et al., 2001). Thus, consistent with the amyloid cascade hypothesis (Hardy and Higgins, 1992), the authors demonstrate that an early pathological species of Aβ is sufficient to trigger an AD-like disease cascade in a novel nonhuman primate model of sAD.

Over the past two decades, reports have suggested that cognitive decline in AD patients is not proportional to Aβ plaque load but does correlate with increases in soluble oligomeric forms of Aβ (Wang et al., 1999). Moreover, the deposition of Aβ in spatiotemporally distinct brain regions is thought to occur in a progressive, spreading manner (Thal et al., 2008). However, much of the evidence for these assertions has come from using AD transgenic mouse models. In their paper, Forny-Germano et al. (2014) found that one injection of 100 μg of AβOs, prepared from synthetic Aβ_1−42_ peptide, every three days for twenty-four days was sufficient to induce AD-like pathology in macaques aged nine and sixteen years. They showed that AβOs distributed and induced pathology in multiple brain regions, including the neocortex, hippocampus, striatum, and thalamus (Forny-Germano et al., 2014, their Figure 3). The authors propose that such progression represents an AD-like intermediary stage of pathology, but whether their findings are the result of the diffusion of solution throughout the brain or the propagation of a disease mechanism remains unclear.

One possible way of addressing this would be to inject an oligomeric random peptide, conjugated to a small peptide tag (such as poly-histidine), and examine whether the pattern of immunostaining is maintained. Interestingly, AβO labeling appeared to be intra- and extracellular as measured by the anti-oligomer NU4 antibody, which does not recognize monomeric Aβ (Forny-Germano et al., 2014, their Figure 2); however, data using APP transgenic mice expressing the E693Δ mutation, which increases Aβ oligomerization without fibrillization, found that intraneuronal AβOs alone were sufficient to induce features of AD pathology (Tomiyama et al., 2010). Additionally, intracellular Aβ_1−42_ aggregation has been found to precede extracellular accumulation in humans (Mori et al., 2002). In short, while the results of Forny-Germano et al. (2014) demonstrate that AβOs distribute and bind to cells in AD-related brain regions, they do not establish whether this represents a universal response to a foreign peptide or to what extent intra vs. extracellular AβO deposition is responsible for their subsequent findings.

Next, using a range of phosphorylated tau antibodies, as well as antibodies recognizing early conformational changes in NFT formation, the authors show by immunostaining, western blotting, and immune-gold electron microscopy that AβO injection leads to tau hyperphosphorylation, truncation, and aggregation (Forny-Germano et al., 2014, their Figure 5). These results are particularly striking owing to their support of the amyloid cascade hypothesis in a species that possesses a far closer approximation of the human brain than rodents. Indeed, cynomolgus macaques are naturally highly resistant to NFT pathology until ≈30 years of age, despite developing Aβ plaques and cerebral amyloidosis at ≈25 years of age and having >99% gene sequence homology to human tau (Heuer et al., 2012). However, these experiments do not elucidate whether AβOs alone are perpetuating alterations in tau. Activation of astrocytes and microglia occurs in this model, and neuroinflammation might contribute more to tau pathology in the disease cascade than is currently recognized. In fact, Aβ-induced astrocyte and microglial-derived soluble factors have been implicated in neuronal tau pathology (Metcalfe and Figueiredo-Pereira, 2010), as well as synapse loss (Eroglu and Barres, 2010), suggesting glial activation may also participate in the observed decrease in synaptophysin and PSD-95 protein expression (Forny-Germano et al., 2014, their Figure 11). Consequently, it may prove useful to compare the relative contribution of AβO deposition, glial activation, and tau pathology to synapse loss between this model and existing transgenic mouse models (Figure 1). It will be crucial to determine the AβO-induced sequence of events in this model to assess what cellular and/or molecular components are directly modulating tau.

**Figure 1:**
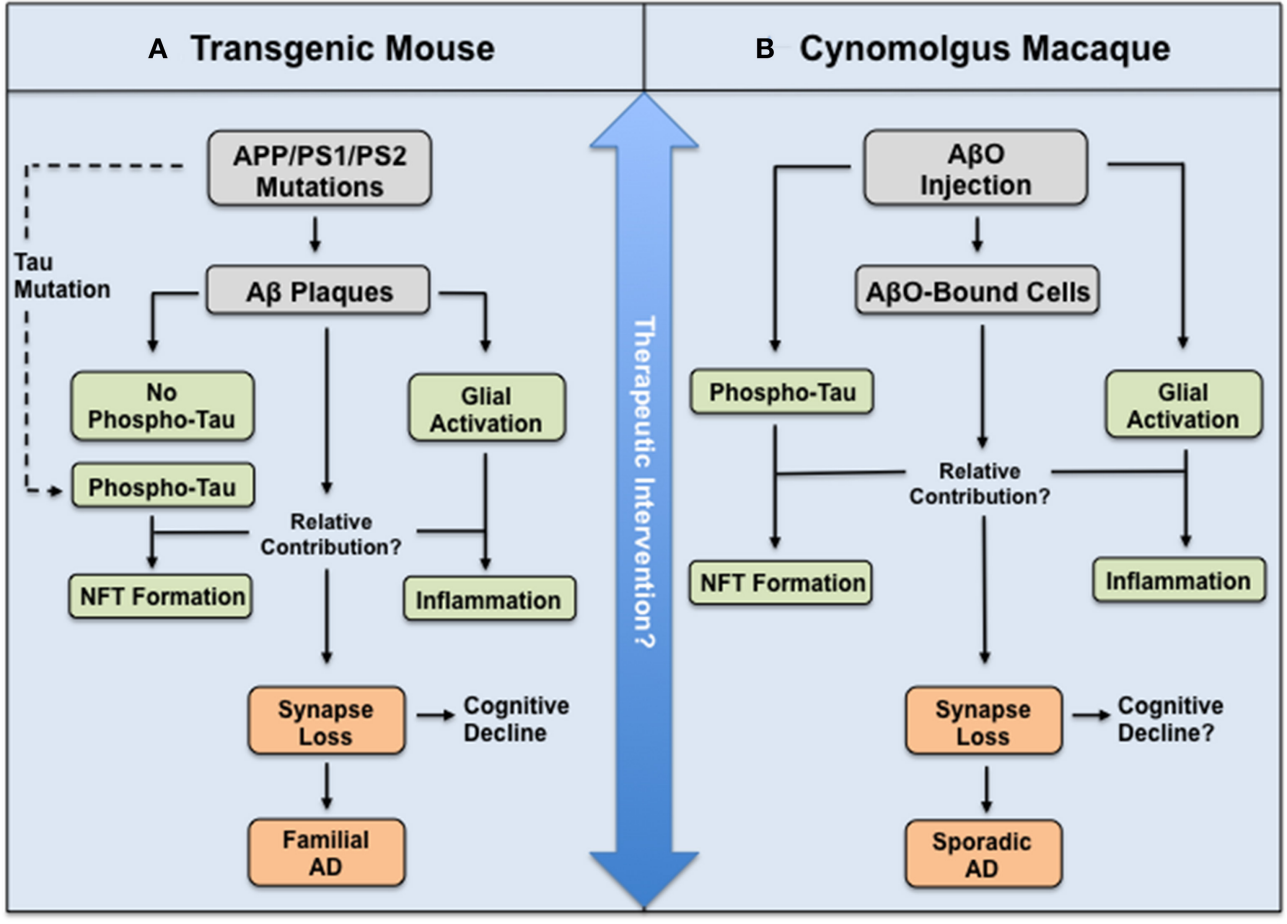
**Illustration depicting relationship between mouse and macaque models of AD in relation to the amyloid cascade hypothesis. (A)** Transgenic mouse and **(B)** Cynomolgus macaque model used by Forny-Germano et al. (2014). Both animals display AD-like pathology and synapse loss, yet the routes and molecular mechanisms underlying this differ to ultimately model either fAD or sAD. However, these differences may prove advantageous to test the efficacy of therapeutic intervention at different stages in the disease cascade.

Like many studies using transgenic mice, the authors report no neuronal cell death in the macaque brain—measured by TUNEL staining of the frontal cortex and amygdala (Forny-Germano et al., 2014, their Figure 10). To confirm this, it would be necessary to examine caspase activation in their tissue sections, for one could surmise that such a large quantity of injected foreign protein (100 μg of AβOs) would cause some degree of neuronal fallout. This finding represents a wider problem for AD animal models, in which chronic exposure to Aβ often does not trigger cell death. Human AD is characterized by widespread neuronal loss; therefore, it would be prudent to test whether long-term exposure of AβOs eventually generates this hallmark feature of AD neuropathology. If it does, one can imagine devising reversibility experiments aimed at inhibiting pathogenesis before an identifiable critical threshold for neuronal death.

Future studies should also explore whether these macaques show behavioral and/or cognitive impairment. AD transgenic mice display robust and well-established deficits in memory-related task, such as the Morris water maze (MWM), but significantly less data exists showing cognitive decline in primate models of AD. This is due in part to the limited numbers of animals studied and the long time period required for them to reach maturity. Plaque density has not been found to correlate with cognitive decline in the closely related rhesus macaques, but studies have shown that recognition memory decline begins during the late teenage years, when increased Aβ is seen in the medial temporal lobe, and progresses concomitantly with increased Aβ deposition in the frontal cortex during the animals' mid-twenties (Heuer et al., 2012). Experiments examining whether AβOs are related to this process would be particularly informative. In addition, it would be interesting to see if the results of Forny-Germano et al. (2014) are the same in male cynomolgus macaques. The authors used seven females for their experiments, and male-female differences might be important considering that in rhesus macaques biosenescence occurs around the same age at which menopause becomes apparent (≈25 years); and notably, reports in AD patients suggest that estrogen may be neuroprotective (Correia et al., 2010).

In closing, Forny-Germano et al. (2014) propose a powerful and unique animal model that recapitulates core features of AD neuropathology in a species biologically close to humans. This may offer a novel research platform for future mechanistic studies aimed at shedding more light on the amyloid cascade hypothesis, as well as testing novel diagnostic and therapeutic agents targeting oligomeric Aβ, hyperphosphorylated tau, and/or neuroinflammation in sporadic AD. Key questions for future studies include: (1) What are the molecular signals underlying AβO-induced tau pathology and synapse loss? (2) Does chronic exposure of AβOs in macaques lead to neuronal loss and/or cognitive impairment? (3) At what stage in the disease cascade would therapeutic intervention provide the most benefit? Experiments aimed at addressing these questions will be helpful to further characterize and explore this model.

## Conflict of interest statement

The authors declare that the research was conducted in the absence of any commercial or financial relationships that could be construed as a potential conflict of interest.
